# Neurological and Neuropsychiatric Manifestations of Pediatric Inflammatory Multisystem Syndrome (PIMS/MIS-C): A Narrative Review

**DOI:** 10.3390/jpm16090461

**Published:** 2026-08-31

**Authors:** Wiktor Śliwiński, Weronika Pura, Dominika Matecka, Mateusz Kosowski, Daniel Chołuj, Jakub Marciniak, Jakub Mazur, Karolina Zarówna, Laavanya Damodaran, Natalia Szejko

**Affiliations:** 1 The Independent Public Healthcare Institution of the Ministry of the Interior and Administration in Lodz, Północna 42, 91-425 Lodz, Poland; wiktor.sliwinski@stud.umed.lodz.pl (W.Ś.); weronika.pura@stud.umed.lodz.pl (W.P.); daniel.choluj@stud.umed.lodz.pl (D.C.); 2 Jozef Strus Municipal Hospital, A Multispecialty Medical Centre in Poznan Szwajcarska Street, 61-285 Poznan, Poland; dominika.matecka@stud.umed.lodz.pl; 3 Independent Public Healthcare Institution in Kolo, 25 Ksiecia Jozefa Poniatowskiego Street, 62-600 Kolo, Poland; mateusz.kosowski@stud.umed.lodz.pl; 4 University Clinical Hospital No. 2 of the Medical University of Łodz, 113 Zeromskiego Street, 90-549 Lodz, Poland; jakub.marciniak@stud.umed.lodz.pl; 5 Voivodeship Combined Hospital in Kielce, 45 Grunwaldzka Street, 25-736 Kielce, Poland; jakub.mazur@stud.umed.lodz.pl; 6 District Hospital Complex in Olesnica, 1 Armii Krajowej Street, 56-400 Olesnica, Poland; karolina.zarowna@stud.umed.lodz.pl; 7 Birmingham Children’s Hospital, Birmingham B4 6NH, UK; 8 Clinic of Psychiatry, Social Psychiatry and Psychotherapy, Hannover Medical School, 30625 Hannover, Germany; 9 Department of Child Psychiatry, Babinski Hospital, 91-229 Lodz, Poland; 10 Department of Health Sociology, Education and Medical Communication, Institute of Mother and Child, 01-211 Warsaw, Poland

**Keywords:** PIMS/MIS-C * SARS-CoV-2, pediatric neurology, neuropsychiatric manifestations, systemic hyperinflammation, encephalopathy, post-infectious complications

## Abstract

**Background:** Pediatric inflammatory multisystem syndrome (PIMS), also referred to as multisystem inflammatory syndrome in children (MIS-C), is a rare but serious post-infectious complication of severe acute respiratory syndrome coronavirus 2 (SARS-CoV-2) infection characterized by systemic hyperinflammation and multiorgan involvement. New-onset neurological and psychiatric symptoms have emerged as critical clinical features, occurring in approximately 12–27% of pediatric patients aged 2–15 years (median age, 10 years) and often indicating a more severe disease course. This narrative review aims to provide a comprehensive overview of the current literature regarding the neurological and psychiatric manifestations of PIMS/MIS-C, focusing on epidemiology, pathophysiology, clinical presentation, neuroimaging findings, biomarkers, treatment strategies, and outcomes. **Methods:** A narrative literature review was conducted to synthesize current evidence on the neurological and psychiatric spectrum of PIMS/MIS-C. Evaluated parameters included clinical presentations ranging from common symptoms (such as headache, encephalopathy, altered mental status, and seizures) to rare complications (such as ischemic stroke, acute disseminated encephalomyelitis, Guillain–Barré syndrome, and cerebral edema), alongside associated psychiatric disturbances, diagnostic findings, and therapeutic approaches. **Results:** Neurological and psychiatric manifestations significantly impact the clinical trajectory of PIMS/MIS-C. Acute symptoms include headache, encephalopathy, seizures, and psychiatric disturbances like behavioral changes, hallucinations, delirium, anxiety, and sleep disorders. Neuroimaging in many cases reveals reversible lesions of the splenium of the corpus callosum, while electroencephalography typically demonstrates diffuse slowing consistent with encephalopathy. Early recognition and prompt administration of immunomodulatory therapy—primarily intravenous immunoglobulin and corticosteroids—correlate with favorable neurological recovery in the majority of patients. However, current evidence remains largely observational and heterogeneous, precluding definitive causal conclusions regarding this treatment–outcome relationship. Affected children more frequently require intensive care unit admission and remain at risk for persistent cognitive, behavioral, and psychiatric sequelae. **Conclusions:** Neurological and psychiatric complications in PIMS/MIS-C are clinically significant indicators of disease severity that require vigilant monitoring and early immunomodulatory intervention. Continued multidisciplinary follow-up and prospective studies are essential to elucidate the long-term neurodevelopmental and psychiatric consequences and to optimize therapeutic strategies for these patients.

## 1. Introduction

Although coronavirus disease 2019 (COVID-19) generally follows a mild clinical course in children with a median age between 7 and 10 years, a small proportion develop a severe post-infectious hyperinflammatory condition known as Pediatric Inflammatory Multisystem Syndrome (PIMS), also referred to internationally as Multisystem Inflammatory Syndrome in Children (MIS-C). First recognized during the early stages of the COVID-19 pandemic, PIMS/MIS-C typically develops 2–6 weeks after exposure to severe acute respiratory syndrome coronavirus 2 (SARS-CoV-2) and is characterized by persistent fever, systemic inflammation, and dysfunction of multiple organ systems, including the cardiovascular, gastrointestinal, mucocutaneous, hematologic, and neurological systems. According to the 2023 CSTE/CDC position statement, Multisystem Inflammatory Syndrome in Children (MIS-C) is defined as an illness in individuals under 21 years of age presenting without a more likely alternative diagnosis—such as Kawasaki disease—characterized by fever, hospitalization-level severity or death, elevated C-reactive protein, and new-onset manifestations in at least two organ systems, accompanied by laboratory evidence of recent SARS-CoV-2 infection or an epidemiologic link to a COVID-19 case within sixty days prior to hospitalization [1]. Although uncommon, PIMS/MIS-C represents one of the most severe pediatric complications associated with SARS-CoV-2 infection and frequently requires hospitalization and multidisciplinary management [2].

The pathogenesis of PIMS/MIS-C differs substantially from that of acute COVID-19. Rather than resulting from direct viral cytotoxicity, the syndrome is believed to arise from an exaggerated immune response triggered by previous SARS-CoV-2 infection in genetically susceptible individuals. Dysregulated activation of innate and adaptive immune pathways, excessive cytokine production, endothelial dysfunction, complement activation, and immune-mediated tissue injury collectively contribute to the multisystem manifestations observed in affected children. These inflammatory mechanisms also appear to play a central role in central and peripheral nervous system involvement, making neurological complications an increasingly important focus of clinical and translational research [2,3,4,5,6,7,8,9].

Neurological and psychiatric manifestations have emerged as significant components of the clinical spectrum of PIMS/MIS-C [10,11]. Reported neurological abnormalities range from relatively common symptoms, such as headache, altered mental status, encephalopathy, irritability, and seizures, to uncommon but potentially life-threatening complications including ischemic stroke, acute disseminated encephalomyelitis, Guillain–Barré syndrome, cerebral edema, and other neuroinflammatory disorders [10,11,12,13,14,15,16,17,18,19,20,21]. Psychiatric manifestations—including behavioral disturbances, hallucinations, delirium, anxiety, mood changes, and sleep disorders—have also been increasingly recognized during both the acute illness and the recovery period [11,14,17,22,23,24,25]. Importantly, children with neurological involvement frequently exhibit more severe systemic inflammation, are more likely to require intensive care support, and may experience persistent cognitive or neuropsychiatric sequelae after hospital discharge [11].

While MIS-C represented a significant public health challenge in the early pandemic, its incidence has substantially declined in subsequent years. This shift is attributed to the evolution of SARS-CoV-2 variants, higher population-level immunity from prior infections, and the widespread adoption of pediatric COVID-19 vaccination, which has been shown to significantly reduce the risk of MIS-C in children. Despite this decrease in incidence, MIS-C remains a clinically relevant diagnosis, particularly in unvaccinated or vulnerable populations, where it frequently necessitates intensive care and multidisciplinary management.

Over the past few years, a rapidly expanding body of literature has improved our understanding of neurological complications associated with PIMS/MIS-C. However, considerable heterogeneity remains regarding the reported prevalence of neurological manifestations, underlying pathogenic mechanisms, neuroimaging findings, treatment strategies, and long-term outcomes. Differences in diagnostic criteria, study design, patient populations, and definitions of neurological involvement have contributed to variability across published studies, highlighting the need for an integrated overview of the available evidence.

The aim of this narrative review is to summarize the current knowledge regarding the neurological and psychiatric manifestations of PIMS/MIS-C. We discuss the epidemiology, immunopathogenesis, clinical presentation, neuroimaging findings, inflammatory biomarkers, therapeutic approaches, and neurological outcomes associated with the syndrome. Furthermore, we highlight current knowledge gaps and future research priorities to improve the prevention, recognition, management, and long-term follow-up of children affected by this complex multisystem inflammatory disorder. By integrating a structured differential diagnosis and a practical clinical algorithm, the review helps clinicians navigate complex diagnostic overlaps—such as distinguishing MIS-C from acute SARS-CoV-2, bacterial or viral CNS infections, toxic shock, Kawasaki disease, autoimmune encephalitis, ADEM, stroke, or medication-related delirium. Furthermore, it breaks valuable new ground by addressing the comparatively underexplored neuropsychiatric and psychological sequelae—including delirium, hallucinations, anxiety, emotional dysregulation, sleep disturbances, and post-intensive care syndrome (PICS-p)—shifting the clinical paradigm from acute survival to holistic, long-term functional recovery.

## 2. Materials and Methods

A comprehensive narrative literature review was conducted to synthesize current evidence on the neurological and psychiatric spectrum of PIMS/MIS-C. We performed a structured search of the PubMed, Scopus, Web of Science, and Frontiers databases for articles published between January 2020 and August 2026. The search strategy utilized a combination of Medical Subject Headings (MeSH) and free-text terms including “PIMS-TS,” “MIS-C,” “neurological manifestations,” “neuroinflammation,” “psychiatric symptoms,” and “SARS-CoV-2.” We included studies involving children and adolescents (aged <21 years) that adopted standardized diagnostic criteria for PIMS/MIS-C, primarily those aligned with the CDC/CSTE (2023) or WHO definitions. Eligible study designs encompassed systematic reviews, meta-analyses, prospective and retrospective cohort studies, case series, and individual case reports. Language restrictions were applied to English-language publications. Exclusion criteria included conference abstracts lacking full data, editorial letters, and studies focusing exclusively on adult populations. The reviewed literature was classified into three evidence classes: (1) direct MIS-C evidence focusing specifically on patients meeting diagnostic criteria for the syndrome; (2) mixed pediatric SARS-CoV-2/MIS-C evidence capturing studies with overlapping acute COVID-19 and hyperinflammatory cohorts; and (3) extrapolated evidence regarding broader neuroinflammatory and immune-mediated mechanisms. References were selected based on their relevance to the clinical, diagnostic, and pathophysiological themes of the review. Data extraction focused on clinical presentations (e.g., encephalopathy, seizures, headache, ischemic stroke, acute disseminated encephalomyelitis), psychiatric disturbances (e.g., delirium, behavioral changes, anxiety), diagnostic findings (neuroimaging and EEG abnormalities), and immunomodulatory treatment strategies. This synthesis was designed to bridge knowledge gaps between acute neurological presentation and long-term neurodevelopmental outcomes, following a multidisciplinary approach consistent with current pediatric intensive care and neurology guidelines.

## 3. Narrative Synthesis of Immunopathogenesis of PIMS/MIS-C

The pathogenesis of PIMS/MIS-C, is incompletely understood but is widely considered to result from an exaggerated post-infectious immune response following infection with severe acute respiratory syndrome coronavirus 2 (SARS-CoV-2). Unlike acute COVID-19, in which tissue injury is primarily associated with viral replication, PIMS/MIS-C typically develops 2–6 weeks after infection, when viral replication has largely resolved [2]. This delayed onset supports the hypothesis that immune dysregulation, rather than direct viral cytotoxicity, is the principal driver of systemic inflammation and multiorgan dysfunction. Although the precise mechanisms remain under investigation, accumulating evidence suggests that interactions among genetic susceptibility, dysregulated immune activation, endothelial injury, and thromboinflammatory pathways contribute to the heterogeneous clinical manifestations observed in affected children [4,7].

### 3.1. Established Pathogenic Mechanisms

Immune dysregulation is a hallmark of PIMS/MIS-C. The syndrome is characterized by profound activation of both innate and adaptive immune responses, resulting in excessive production of pro-inflammatory cytokines and chemokines. Elevated serum concentrations of interleukin (IL)-1β, IL-6, IL-8, IL-10, IL-17, tumor necrosis factor-α (TNF-α), interferon-γ, and granulocyte-macrophage colony-stimulating factor have been consistently reported and correlate with disease severity. These mediators promote leukocyte recruitment, endothelial activation, vascular permeability, and amplification of systemic inflammation [7].

Among these cytokines, IL-1β and IL-6 appear to play particularly important roles in sustaining the hyperinflammatory response, providing the biological rationale for the therapeutic use of IL-1 receptor antagonists (anakinra) and IL-6 inhibitors (tocilizumab) in selected patients with refractory disease [26,27] Elevated IL-10 concentrations, despite their anti-inflammatory properties, likely reflect a compensatory response to overwhelming immune activation rather than effective immune regulation [28].

Endothelial dysfunction represents another fundamental mechanism underlying PIMS/MIS-C. Pro-inflammatory cytokines activate vascular endothelial cells, increasing the expression of adhesion molecules, promoting leukocyte infiltration, and disrupting vascular homeostasis. Endothelial injury is accompanied by activation of the complement cascade, particularly the terminal complement pathway, resulting in additional inflammatory amplification and microvascular damage [9].

Simultaneously, inflammatory activation promotes a prothrombotic state characterized by platelet activation, increased fibrinogen concentrations, elevated D-dimer levels, and excessive thrombin generation. This process, commonly referred to as immunothrombosis, may integrate inflammatory pathways with the coagulation cascade and contribute to the formation of microvascular thrombi within multiple organs. Cerebral microvascular injury and impaired cerebral perfusion may therefore contribute to neurological manifestations such as encephalopathy, ischemic stroke, and diffuse cerebral dysfunction. The frequent observation of markedly elevated D-dimer concentrations and coagulation abnormalities has consequently led to the routine use of thromboprophylaxis in selected high-risk patients [11,29].

#### 3.1.1. Emerging Hypotheses and Research Frontiers

Several complex mechanisms have been proposed, yet their contribution to MIS-C neurological pathology remains under investigation. Current evidence suggests that neurological manifestations of PIMS/MIS-C are predominantly immune-mediated rather than caused by direct invasion of the central nervous system by SARS-CoV-2 [3]. Systemic inflammation induces endothelial activation within cerebral vessels, resulting in increased permeability of the blood–brain barrier. This disruption facilitates the entry of inflammatory cytokines, activated immune cells, and circulating antibodies into the central nervous system, thereby promoting neuroinflammation. Within the brain, activation of resident microglia and astrocytes further amplifies the inflammatory cascade through local cytokine production and oxidative stress [30]. These processes may impair neuronal function without causing irreversible structural injury, which may explain why many children develop encephalopathy despite normal or only subtle neuroimaging findings. In severe cases, however, sustained neuroinflammation may contribute to cerebral edema, demyelinating syndromes, or cerebrovascular complications [26,31,32]. Emerging evidence indicates that adaptive immune responses may also contribute to disease pathogenesis. Clinical and molecular studies have identified a profound expansion of TRBV11-2 T-cell clones in MIS-C patients, which correlates significantly with disease severity and serum cytokine levels. Furthermore, this clonal expansion is associated with a shared genetic predisposition involving the HLA class I alleles A02, B35, and C04 [33]. Genetic diversity may partially explain the marked heterogeneity in disease severity, organ involvement, and response to immunomodulatory therapy observed among affected children.

#### 3.1.2. Hypothetical Pathways: Emerging Research Frontiers

Several studies have identified autoantibodies directed against endothelial, cardiac, gastrointestinal, and immune regulatory proteins, suggesting that molecular mimicry following SARS-CoV-2 infection may trigger autoimmune mechanisms in susceptible individuals [34]. Although no disease-specific neuronal autoantibody has been consistently identified, immune-mediated neuronal dysfunction remains a plausible explanation for neurological and psychiatric manifestations occurring in the absence of detectable viral infection within the cerebrospinal fluid. Abnormal activation of T lymphocytes, expansion of activated CD8^+^ T cells, and disturbances in B-cell maturation have additionally been reported, supporting the concept that persistent immune dysregulation extends beyond the acute inflammatory phase [4,6]. Further investigation of these adaptive immune pathways may facilitate the identification of biomarkers capable of predicting neurological complications and treatment response. The rarity of PIMS/MIS-C despite widespread exposure to SARS-CoV-2 strongly suggests an underlying genetic predisposition. Variants affecting interferon signaling, human leukocyte antigen (HLA) haplotypes, inflammasome activation, complement regulation, and cytokine signaling pathways have been proposed as potential susceptibility factors, although their clinical significance remains under investigation [35]. Emerging evidence also suggests a potential role for nutritional factors in modulating disease severity. Furthermore, in a cohort of 63 affected children (median age 10.3 years), baseline Vitamin D levels inversely correlated with peak CRP (r = −0.34, *p* = 0.007) and the duration of vasoactive support (r = −0.34, *p* = 0.030), suggesting it may act as a modifier of disease severity [36] From the perspective of personalized medicine, integrating genomic data with clinical characteristics, inflammatory biomarkers, and advanced neuroimaging may enable individualized risk stratification and earlier identification of patients at increased risk of neurological complications. Such approaches may ultimately support biomarker-guided therapeutic decisions and improve long-term neurological outcomes.

**Figure 1:**
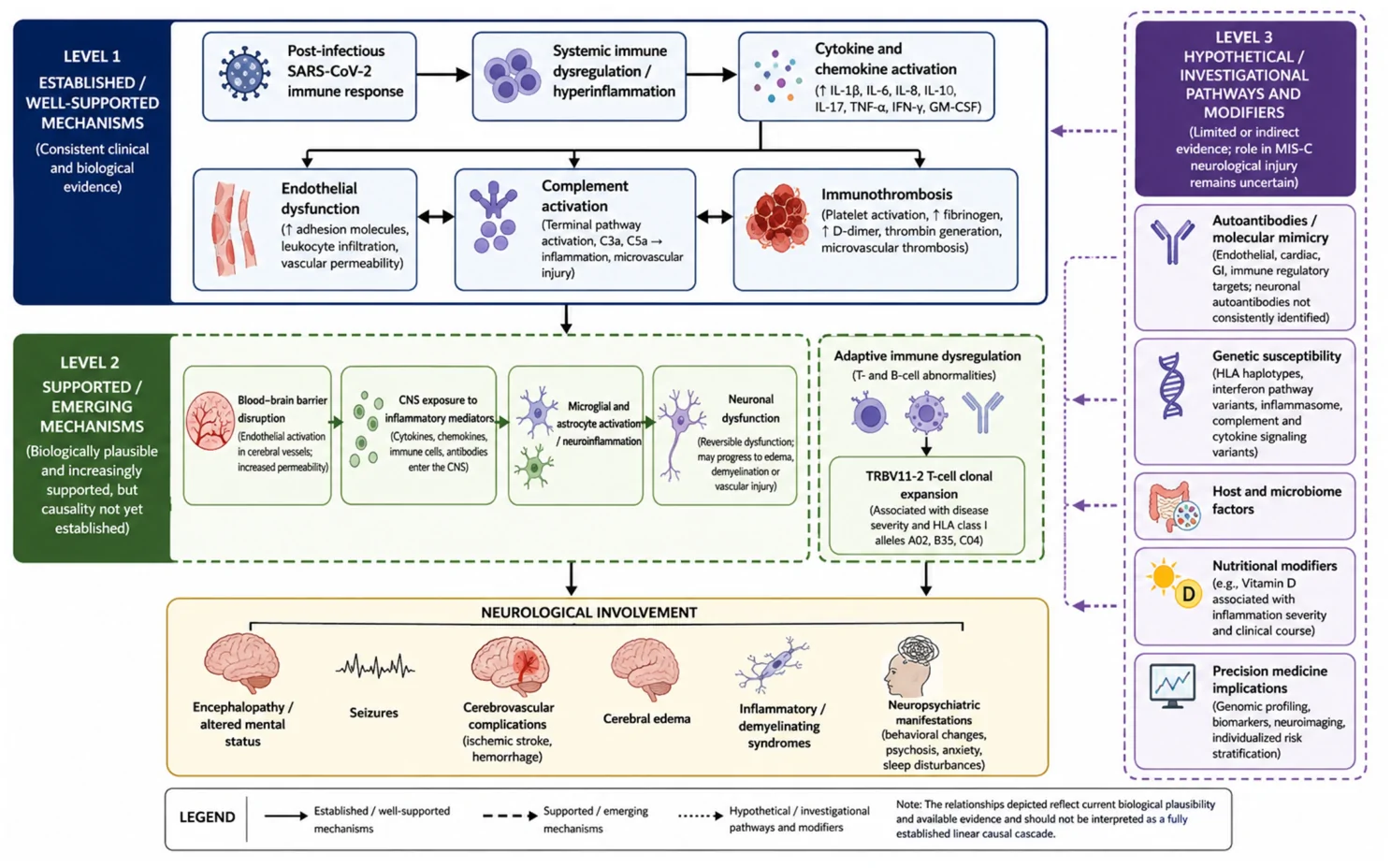
Proposed mechanisms underlying neurological involvement in PIMS/MIS-C stratified according to the current level of evidence. The figure illustrates a hierarchical model of potential pathways contributing to neurological manifestations of PIMS/MIS-C. Established or well-supported mechanisms include post-infectious immune dysregulation, systemic hyperinflammation and cytokine activation, endothelial dysfunction, complement activation, and immunothrombosis. Supported but emerging mechanisms include blood–brain barrier disruption, CNS exposure to inflammatory mediators, microglial and astrocytic activation, neuronal dysfunction, and adaptive immune abnormalities, including TRBV11-2 T-cell clonal expansion. Hypothetical or investigational pathways and modifiers include autoantibody-mediated mechanisms and molecular mimicry, genetic susceptibility, host-related factors, and nutritional modifiers such as vitamin D. Solid, dashed, and dotted lines indicate established/well-supported, supported/emerging, and hypothetical/investigational relationships, respectively. The depicted pathways reflect the current strength of available evidence and biological plausibility and should not be interpreted as a fully established linear causal cascade.

Current evidence indicates that neurological injury in MIS-C evolves through a structured pathophysiological cascade (Figure 1). Elucidating these mechanisms is essential for identifying reliable biomarkers, improving risk stratification, and advancing personalized therapeutic approaches for children with neurological involvement.

### 3.2. Epidemiology and Risk Factors for Neurological Involvement

The epidemiology of PIMS/MIS-C affects predominantly school-aged children and adolescents, with a median age ranging from 7 to 10 years [37,38]. Several large international cohorts have demonstrated a slight male predominance among hospitalized children, although sex-related differences in neurological manifestations remain inconsistent [39,40]. Whether biological sex influences susceptibility to neuroinflammation or immune dysregulation has not yet been established. Marked ethnic disparities have also been reported. Higher incidences of PIMS/MIS-C have been observed among hospitalized children of African, Hispanic/Latino, and South Asian ancestry compared with White pediatric populations [38]. These differences are likely multifactorial and may reflect interactions between genetic susceptibility, socioeconomic determinants, healthcare access, environmental exposures, and differences in SARS-CoV-2 transmission rather than ethnicity alone. Nevertheless, these observations support continued investigation of host genetic factors that may influence disease phenotype and neurological complications.

Approximately 22–47% of pediatric patients in the US experience neurological symptoms related to COVID-19 infection in general [41]. Neurological involvement is increasingly recognized as a significant component of the clinical spectrum of PIMS/MIS-C. One study involving 161 hospitalized children demonstrated that patients with PIMS-TS (*n* = 50) presented neurological symptoms significantly more often than those with acute COVID-19 (*n* = 161) (28% vs. 10.6%) [38]. Although reported frequencies vary considerably because of differences in study design, diagnostic criteria, and definitions of neurological involvement, new-onset neurological manifestations have been described in approximately 12–27% of hospitalized children with PIMS/MIS-C and in 27% of children with PIMS/MIS-C in general [10,11].

Children presenting with encephalopathy, seizures, or altered consciousness are more likely to require admission to a pediatric intensive care unit (PICU), vasopressor support, mechanical ventilation, and prolonged hospitalization [42]. Cardiac involvement, including myocarditis, ventricular dysfunction, coronary artery abnormalities, arrhythmias, and cardiogenic shock, represents one of the strongest indicators of severe PIMS/MIS-C [43].

Although most children recover without permanent neurological deficits, a subset develops persistent cognitive, behavioral, or psychiatric sequelae requiring long-term follow-up [20,23]. Neuroimaging abnormalities, particularly diffuse cerebral edema, ischemic lesions, and inflammatory white matter changes, may also predict a more complicated clinical course [14,26]. Given the heterogeneity of PIMS/MIS-C, early identification of children at increased risk of neurological complications remains a major clinical priority. Integrating demographic characteristics, clinical presentation, inflammatory biomarkers, cardiac involvement, neuroimaging findings, and emerging molecular biomarkers may facilitate individualized risk stratification and personalized therapeutic decision-making.

### 3.3. Neurological Manifestations

The spectrum ranges from mild, self-limiting symptoms to severe, life-threatening neurological complications involving both the central and peripheral nervous systems. Importantly, neurological symptoms often coincide with severe systemic inflammation and cardiovascular dysfunction, suggesting that immune-mediated injury, endothelial dysfunction, and impaired cerebral perfusion collectively contribute to nervous system involvement. Neurological involvement significantly worsens the clinical trajectory of MIS-C. In a large cohort, severe neurological manifestations were observed in 24.8% of patients with MIS-C, a higher prevalence than in acute SARS-CoV-2 infection (18.0%). Crucially, these manifestations are strong predictors of long-term impairment; survivors with severe neurological involvement were significantly more likely to experience new neurocognitive or functional morbidity at hospital discharge compared to those without (28.0% vs. 15.5%; *p* = 0.002). After adjusting for other risk factors, patients with MIS-C presenting with severe neurological symptoms faced over twofold higher odds (OR 2.18; 95% CI, 1.22–3.89) of persistent morbidity, underscoring the critical need for vigilant neurological monitoring and follow-up [14].

#### 3.3.1. Common Neurological Manifestations

Headache is the most frequently reported neurological symptom in PIMS/MIS-C, affecting approximately one-third to one-half of patients [11,12,17,18,22,23,26,42,44]. It is typically diffuse, persistent, and often accompanies fever and elevated inflammatory markers. Although the precise mechanism remains uncertain, headache is likely multifactorial, resulting from systemic cytokine release, endothelial activation, meningeal irritation, and neuroinflammation. In most children, symptoms improve rapidly following immunomodulatory therapy, supporting an inflammatory rather than structural ethology.

Encephalopathy is among the most characteristic neurological manifestations of PIMS/MIS-C and represents an important marker of severe disease [10,12,13,14,22,26,42,44]. Clinical presentation ranges from mild somnolence and reduced attention to profound impairment of consciousness requiring intensive care support. Neuroimaging is frequently normal or demonstrates transient abnormalities, while electroencephalography (EEG) commonly reveals diffuse background slowing consistent with generalized cerebral dysfunction. The pathogenesis is believed to involve systemic inflammation, cytokine-mediated neurotoxicity, endothelial dysfunction, blood–brain barrier disruption, and cerebral hypoperfusion rather than direct viral invasion of the central nervous system.

Seizures are among the most serious neurological complications of PIMS/MIS-C and may be either generalized or focal. They frequently occur in children with severe encephalopathy and are often associated with markedly elevated inflammatory markers and multiorgan failure [11,12,14,17,20,22,23,26,42,44,45]. Although most seizures are isolated and respond to standard antiepileptic therapy together with immunomodulation, rare cases of status epilepticus have been reported, necessitating aggressive intensive care management. Continuous EEG monitoring should be considered in patients with persistent altered consciousness because non-convulsive seizures may remain clinically unrecognized.

Acute confusion, disorientation, impaired concentration, and fluctuating consciousness are frequently observed during the hyperinflammatory phase of the illness [23,45]. These symptoms often occur in conjunction with encephalopathy and correlate with elevated inflammatory markers and multiorgan dysfunction. Early recognition is essential because deterioration in mental status may indicate evolving cerebral edema, seizures, or other severe neurological complications.

Irritability is particularly common in younger children and may represent one of the earliest neurological manifestations of PIMS/MIS-C [23]. Behavioral disturbances include agitation, emotional lability, excessive crying, and decreased interaction with caregivers [11,20,23,26,44,45]. Although nonspecific, these symptoms likely reflect diffuse neuroinflammation and should prompt careful neurological assessment, especially when accompanied by altered consciousness or persistent fever.

Dizziness and vertigo have been described less frequently but may occur during both the acute inflammatory phase and early recovery [23,42]. Proposed mechanisms include autonomic dysfunction, systemic hypotension, vestibular involvement, and impaired cerebral perfusion secondary to cardiovascular dysfunction. Although generally transient, persistent symptoms warrant further neurological evaluation.

Ischemic stroke represents a rare but devastating complication of PIMS/MIS-C. Multiple mechanisms have been proposed, including endothelial injury, complement activation, hypercoagulability, immunothrombosis, cardiac dysfunction with embolic events, and inflammatory vasculopathy. Hemorrhagic stroke has been reported considerably less frequently but may occur secondary to severe coagulopathy or vascular injury [14,17,19,26,42]. Prompt neuroimaging and multidisciplinary management involving pediatric neurologists, intensivists, cardiologists, and hematologists are essential to optimize outcomes. Several children present with clinical signs suggestive of meningitis, including neck stiffness, photophobia, headache, and vomiting [10,14,19,26,42]. Cerebrospinal fluid analysis is usually sterile and demonstrates either normal findings or only mild inflammatory changes, supporting an immune-mediated process rather than bacterial or viral central nervous system infection [12,13]. Consequently, aseptic meningitis should be considered within the neurological spectrum of PIMS/MIS-C once infectious causes have been excluded.

#### 3.3.2. Uncommon Severe Neurological Complications and Inflammatory Syndromes

Diffuse cerebral edema is an uncommon yet life-threatening manifestation that typically occurs in critically ill children with severe hyperinflammation and shock. Clinical deterioration may be rapid, presenting with progressive encephalopathy, seizures, abnormal pupillary responses, and signs of increased intracranial pressure. Neuroimaging demonstrates diffuse cerebral swelling, and management requires immediate neurocritical care alongside aggressive immunomodulatory treatment [10,11,12,13,14,15,16,17,18,19,20,21,22].

ADEM is an immune-mediated demyelinating disorder affecting the central nervous system that has been reported in association with PIMS/MIS-C [19]. Patients typically present with encephalopathy accompanied by multifocal neurological deficits, including weakness, ataxia, cranial nerve involvement, or visual disturbances. Extrapontine central myelinolysis was also present in one patient [32]. Magnetic resonance imaging (MRI) characteristically demonstrates multifocal bilateral white matter lesions consistent with inflammatory demyelination. High-dose corticosteroids remain the cornerstone of treatment, with intravenous immunoglobulin or plasma exchange reserved for refractory cases [46].

Guillain–Barré syndrome (GBS) represents the most frequently reported peripheral nervous system complication associated with PIMS/MIS-C. Clinical manifestations include progressive symmetrical limb weakness, diminished or absent tendon reflexes, sensory disturbances, and, in severe cases, respiratory muscle involvement [19,26]. The disorder is presumed to result from autoimmune responses triggered by molecular mimicry following SARS-CoV-2 infection. Intravenous immunoglobulin remains the preferred first-line therapy, and most pediatric patients achieve substantial neurological recovery with timely treatment.

Posterior reversible encephalopathy syndrome (PRES) has been reported sporadically in children with PIMS/MIS-C. Clinical manifestations include headache, visual disturbances, seizures, confusion, and hypertension. MRI typically demonstrates symmetrical vasogenic edema involving the parieto-occipital regions [45,47]. Endothelial dysfunction, impaired cerebral autoregulation, severe inflammation, and blood pressure fluctuations are considered major pathogenic mechanisms. Recognition is important because neurological deficits are usually reversible following appropriate treatment of the underlying inflammatory process and optimization of blood pressure.

Although direct viral encephalitis appears to be uncommon in PIMS/MIS-C, several patients fulfill clinical and radiological criteria for autoimmune or inflammatory encephalitis [10,14,19,26,42]. Cerebrospinal fluid polymerase chain reaction (PCR) testing for SARS-CoV-2 is generally negative, supporting an immune-mediated mechanism. MRI findings are variable and may include cortical, subcortical, or limbic abnormalities. Early immunotherapy is associated with favorable neurological recovery in most reported cases.

#### 3.3.3. Neuropathies Associated with PIMS/MIS-C

Isolated peripheral neuropathies have been described in association with PIMS/MIS-C, although they remain uncommon. Patients may develop sensory deficits, distal weakness, paresthesia, or mononeuropathies resulting from inflammatory injury, ischemia, or prolonged critical illness. Electrophysiological studies may demonstrate axonal or demyelinating patterns, while rehabilitation plays an important role in functional recovery [12,22,44].

Cranial neuropathies are rare but increasingly recognized manifestations of PIMS/MIS-C. Facial nerve palsy is reported most frequently, although oculomotor, abducens, and lower cranial nerve involvement has also been described [17,19,42]. Proposed mechanisms include inflammatory neuritis, microvascular ischemia, and immune-mediated demyelination. Prognosis is generally favorable following prompt immunotherapy, although prolonged neurological follow-up is recommended.

## 4. Psychiatric and Neuropsychological Manifestations

Although the neurological complications of PIMS have been increasingly recognized, psychiatric and neuropsychological manifestations remain comparatively underexplored. Emerging evidence indicates that acute neuropsychiatric symptoms are relatively common and may occur independently or in association with neurological involvement. Their pathogenesis is likely multifactorial, reflecting the combined effects of systemic inflammation, cytokine-mediated neurotoxicity, blood–brain barrier dysfunction, intensive care exposure, psychological stress, and, in some cases, direct consequences of prolonged hospitalization. While most psychiatric symptoms improve following resolution of the acute inflammatory process, persistent cognitive and emotional difficulties have been described in a subset of patients, emphasizing the importance of long-term multidisciplinary follow-up.

Delirium represents one of the most frequently reported acute manifestations in children hospitalized with PIMS/MIS-C, particularly among those requiring intensive care [14,42]. It is characterized by an acute disturbance of attention, awareness, and cognition, often accompanied by fluctuating levels of consciousness. Clinical features include disorientation, impaired concentration, agitation, psychomotor slowing, and altered sleep–wake cycles. The true prevalence remains difficult to ascertain due to the heterogeneity of diagnostic criteria used across pediatric studies. While delirium is often attributed to acute cerebral dysfunction driven by systemic hyperinflammation and metabolic derangements, distinguishing it from primary psychiatric pathology is challenging. Current literature often conflates acute encephalopathy with delirium, highlighting a critical need for standardized pediatric screening tools in future studies to differentiate these conditions. In the acute phase of PIMS/MIS-C, delirium should be clinically interpreted as a manifestation of acute cerebral dysfunction rather than a primary psychiatric disorder. It is crucial to distinguish this state from psychiatric pathology; delirium often overlaps with encephalopathy and is driven by multifactorial insults, including systemic inflammation, excessive cytokine production, endothelial dysfunction, metabolic disturbances, and neuroinflammation. Additional contributing factors include hypoxia, sedative medications, mechanical ventilation, and environmental stress associated with intensive care admission. Hallucinations occurring during the acute phase should be interpreted as symptoms of acute delirium rather than indicative of primary psychiatric illness. Early recognition is essential because pediatric delirium is associated with prolonged hospitalization and adverse neurodevelopmental outcomes.

Visual and auditory hallucinations have been reported in children with severe PIMS/MIS-C, frequently occurring in the context of encephalopathy or delirium [11,12,22,23,26,44]. Less commonly, patients may develop transient psychotic symptoms, including paranoid ideation or disorganized behavior. Although these manifestations are uncommon, they highlight the profound effects of systemic inflammation on cerebral function. Proposed mechanisms include cytokine-mediated alterations in neurotransmitter signaling, blood–brain barrier disruption, and diffuse cortical dysfunction. Most reported cases resolve following immunomodulatory therapy and treatment of the underlying inflammatory syndrome, suggesting that immune-mediated rather than primary psychiatric mechanisms predominate. In pediatric PIMS/MIS-C, episodes of psychosis generally exhibit a favorable response to immunomodulatory therapy with rapid clinical resolution, a pattern reminiscent of adult cases where psychotic features typically persist for up to 90 days. Despite this relatively swift acute recovery, it is important to note that adolescents may be at a higher risk than adults for experiencing psychotic symptoms within the six months following SARS-CoV-2 infection, highlighting the need for prolonged vigilance even in patients who show initial improvement [48].

Anxiety and depressive symptoms have increasingly been recognized during both the acute phase of PIMS/MIS-C and subsequent recovery [23]. Children may experience excessive fear, emotional distress, persistent sadness, loss of interest in usual activities, social withdrawal, and reduced motivation. These symptoms likely reflect an interaction between neurobiological mechanisms—including persistent neuroinflammation and hypothalamic–pituitary–adrenal axis dysregulation—and psychosocial stressors such as hospitalization, separation from caregivers, and concerns regarding serious illness. Although anxiety and depressive symptoms are frequently reported in the subacute phase of PIMS/MIS-C, their clinical trajectory remains a subject of debate. Recent evidence, including a two-year retrospective study, suggests that these manifestations are largely transient, with mood and anxiety incidence returning to baseline levels within two months post-infection [48]. However, these findings should be interpreted with caution; most existing studies lack pre-morbid baseline assessments, making it difficult to differentiate between direct neurobiological sequelae—such as hypothalamic–pituitary–adrenal axis dysregulation—and reactive psychological responses to the trauma of critical illness. This distinction is crucial, as the transient nature of these symptoms may suggest a reactive process rather than a chronic psychiatric disorder, a hypothesis that warrants further longitudinal investigation. Family anxiety and disruption of normal social and educational activities may further contribute to psychological distress. Early psychological assessment and family-centered support should therefore be considered integral components of comprehensive patient care.

Emotional dysregulation is increasingly recognized following recovery from PIMS/MIS-C. Children may exhibit irritability, mood instability, impulsivity, frustration intolerance, emotional lability, and difficulties adapting to routine daily activities [24,25]. These behavioral changes may interfere with family functioning, school performance, and peer relationships. Although the underlying mechanisms remain uncertain, persistent neuroinflammation affecting frontolimbic neural networks, together with psychological trauma associated with critical illness, are considered likely contributors. Recognition of these symptoms is important because they may persist despite apparent physical recovery.

Sleep disturbances are common during hospitalization and may persist for several months after discharge. Reported symptoms include insomnia, fragmented sleep, nightmares, excessive daytime sleepiness, and circadian rhythm disturbances [11,18,23,49]. Sleep disruption may result from persistent inflammation, anxiety, hospitalization, intensive care exposure, medication effects, or autonomic dysfunction. Sleep disturbances may persist in the subacute phase, with studies reporting significant differences or disordered sleep symptoms lasting beyond 60 to 90 days post-discharge [48]. Because sleep plays a critical role in cognitive recovery and emotional regulation, early identification and appropriate management of sleep disorders may improve both neurological and psychological outcomes.

Children requiring pediatric intensive care for severe PIMS/MIS-C are at risk of developing post-intensive care syndrome (PICS), a condition characterized by persistent physical, cognitive, and psychological impairments following critical illness. Neuropsychiatric manifestations include anxiety, depression, impaired attention, memory deficits, sleep disturbances, fatigue, and reduced health-related quality of life [50]. Family members may simultaneously experience significant psychological distress, including symptoms of post-traumatic stress disorder, emphasizing the importance of family-centered rehabilitation programs. Early mobilization, cognitive rehabilitation, and structured psychological follow-up may reduce the long-term burden of PICS.

Overall, psychiatric and neuropsychological manifestations constitute an increasingly recognized dimension of PIMS/MIS-C that extends beyond the acute inflammatory phase. Their clinical significance lies not only in their impact on quality of life but also in their potential influence on neurodevelopment, educational achievement, and psychosocial functioning. A multidisciplinary approach involving pediatric neurologists, psychiatrists, psychologists, intensivists, rehabilitation specialists, and primary care physicians is therefore essential for comprehensive management. From the perspective of personalized medicine, integrating clinical assessment with neuropsychological testing, inflammatory biomarkers, neuroimaging, and long-term follow-up may facilitate early identification of children at risk for persistent neuropsychiatric sequelae and enable individualized rehabilitation strategies.

## 5. Biomarkers and Diagnostic Evaluation

The heterogeneous clinical presentation of Pediatric Inflammatory Multisystem Syndrome (PIMS/MIS-C), also referred to as Multisystem Inflammatory Syndrome in Children (MIS-C), underscores the need for reliable biomarkers capable of identifying patients at increased risk of neurological complications. Although no single laboratory marker is sufficiently sensitive or specific to predict neurological involvement, a combination of clinical, inflammatory, cardiac, and neurological biomarkers, together with neuroimaging and electrophysiological investigations, may improve early diagnosis, disease monitoring, and individualized therapeutic decision-making. From the perspective of personalized medicine, integrating multimodal biomarkers into clinical practice represents an important strategy for optimizing risk stratification and tailoring immunomodulatory treatment.

### 5.1. Laboratory Findings Associated with PIMS/MIS-C

Numerous laboratory abnormalities such as elevated concentrations of C-reactive protein (CRP), ferritin, D-dimer, fibrinogen and procalcitonin reflect the intense systemic inflammatory response characteristic of the syndrome and generally correlate with disease severity [11,29,43,51,52]. Increased levels of cardiac biomarkers, including troponin and B-type natriuretic peptide (BNP) or N-terminal pro-BNP (NT-proBNP), frequently accompany neurological manifestations due to concurrent myocardial injury [11,29,36,43,51,53]. Hematological abnormalities, including lymphopenia, thrombocytopenia, neutrophilia, and hypoalbuminemia, have also been associated with more severe disease [51,53,54]. Elevated concentrations of pro-inflammatory cytokines, particularly IL-6, IL-1β, TNF-α, IL-8, and IL-17, may contribute directly to neuroinflammation through endothelial activation, blood–brain barrier disruption, and activation of resident glial cells [7]. Although these biomarkers currently lack sufficient specificity for routine neurological risk prediction, they provide important insights into disease pathophysiology and may support future biomarker-guided approaches to patient stratification. Emerging neurological biomarkers, including neurofilament light chain (NfL), glial fibrillary acidic protein (GFAP), neuron-specific enolase (NSE), and S100 calcium-binding protein B (S100B), have demonstrated promise as indicators of neuronal and astroglial injury in inflammatory neurological disorders [55]. However, evidence regarding their diagnostic and prognostic value in PIMS/MIS-C remains limited and strictly investigational. Larger prospective studies are required before their routine clinical implementation.

### 5.2. Neurodiagnostic Investigations

Magnetic resonance imaging (MRI) is the preferred neuroimaging modality for evaluating neurological complications in PIMS/MIS-C. MRI findings range from normal examinations to diffuse cerebral edema, ischemic infarction, inflammatory white matter lesions, acute disseminated encephalomyelitis (ADEM), and reversible splenial lesions of the corpus callosum, also referred to as mild encephalitis/encephalopathy with a reversible splenial lesion (MERS) [12,16,19,22,45,49,56]. Despite significant neurological symptoms, many children demonstrate normal MRI findings, supporting the concept of functional neuroinflammation rather than irreversible structural injury.

Electroencephalography (EEG) is recommended in patients presenting with altered mental status, encephalopathy, or seizures. The most common abnormality is generalized diffuse slowing, consistent with global cerebral dysfunction. Less frequently, focal slowing, epileptiform discharges, or non-convulsive status epilepticus may be identified [17,19,22]. EEG is particularly valuable for detecting subclinical seizure activity in critically ill children with persistent encephalopathy.

Lumbar puncture should be considered when central nervous system infection cannot be excluded or inflammatory neurological disorders are suspected. Cerebrospinal fluid (CSF) findings are generally normal or demonstrate mild lymphocytic pleocytosis and modest protein elevation [13]. Importantly, SARS-CoV-2 RNA is rarely detected in CSF, providing further evidence that neurological injury in PIMS/MIS-C is predominantly immune mediated rather than caused by direct viral neuroinvasion. Future studies evaluating inflammatory cytokines, chemokines, neuronal proteins, and autoantibodies in CSF may improve understanding of disease mechanisms.

Evidence regarding positron emission tomography (PET) in pediatric PIMS/MIS-C remains extremely limited. However, reports from autoimmune encephalitis and pediatric post-COVID neurological syndromes suggest that fluorodeoxyglucose PET may detect regional cerebral hypometabolism even in patients with normal MRI findings [57,58]. The role of positron emission tomography (PET) in PIMS/MIS-C is strictly experimental. Because data are extrapolated from autoimmune encephalitis and post-COVID syndromes rather than MIS-C itself, PET is not recommended for routine clinical use and remains confined to research settings investigating persistent neuroinflammation.

### 5.3. Biomarkers for Personalized Risk Stratification

Current evidence suggests that no individual biomarker adequately predicts neurological involvement in PIMS/MIS-C. Instead, combining demographic factors, clinical presentation, laboratory findings, neuroimaging, and emerging molecular biomarkers is likely to provide the greatest diagnostic and prognostic value. A multimodal approach integrating CRP, ferritin, D-dimer, fibrinogen, troponin, BNP, and cytokine profiles with neurological biomarkers such as NfL and GFAP may facilitate early identification of children at increased risk of encephalopathy, seizures, cerebrovascular complications, or persistent neurocognitive impairment.

Future precision medicine strategies are expected to combine circulating biomarkers with advanced neuroimaging, electrophysiological studies, genomic profiling, and machine learning algorithms to generate individualized risk prediction models. Such approaches could support earlier initiation of targeted immunomodulatory therapy, optimize monitoring during hospitalization, and identify children who would benefit from structured neurological and neuropsychological follow-up after recovery. Prospective multicenter studies are required to validate these biomarkers and establish standardized algorithms for personalized management of neurological complications in PIMS/MIS-C.

### 5.4. Differential Diagnosis and Practical Diagnostic Approach

Neurological and psychiatric manifestations in PIMS/MIS-C are heterogeneous and frequently nonspecific. Consequently, the temporal association with SARS-CoV-2 infection should not by itself be considered sufficient to attribute encephalopathy, seizures, behavioral change, focal neurological deficits, or other acute neurological manifestations to PIMS/MIS-C. The diagnosis requires integration of the characteristic systemic inflammatory and multiorgan phenotype with evidence of recent SARS-CoV-2 infection while actively excluding alternative diagnoses [23,59]. This is particularly important because widespread previous SARS-CoV-2 infection and vaccination have reduced the specificity of SARS-CoV-2 seropositivity for the diagnosis of PIMS/MIS-C [60].

Considerable clinical overlap may occur, including fever, systemic inflammation, gastrointestinal symptoms, encephalopathy, and other neurological manifestations. SARS-CoV-2 PCR/antigen testing, the temporal relationship between infection and symptom onset, respiratory involvement, and the broader pattern of organ dysfunction should therefore be interpreted together rather than in isolation [61].

Bacterial sepsis, toxic shock syndrome, and bacterial or viral CNS infection must be excluded early because delayed antimicrobial or antiviral therapy may have serious consequences. Fever, shock, rash, elevated inflammatory markers, altered consciousness, seizures, and meningeal symptoms may occur in both PIMS/MIS-C and severe infection [62,63]. Blood cultures and other microbiological investigations should therefore be obtained according to the clinical presentation, and empirical antimicrobial treatment should not be delayed when invasive bacterial infection is clinically suspected [64]. In children presenting with meningism, seizures, marked encephalopathy, or otherwise unexplained altered consciousness, CSF examination should be considered when clinically safe, including cell count, protein and glucose measurements, bacterial culture, and pathogen-specific PCR testing as indicated. Notably, the generally normal or mildly inflammatory CSF profile and infrequent detection of SARS-CoV-2 RNA in CSF in PIMS/MIS-C support a predominantly immune-mediated rather than directly neuroinvasive mechanism [65].

Kawasaki disease (KD) represents another major inflammatory differential diagnosis because PIMS/MIS-C may present with fever, conjunctival injection, rash, mucosal changes, extremity abnormalities, and coronary artery involvement. However, PIMS/MIS-C generally affects older children and is more frequently associated with prominent gastrointestinal manifestations, myocardial dysfunction, shock, lymphopenia, thrombocytopenia, and neurological involvement. Unlike children with KD, MIS-C patients encompass a broader age distribution, suffer from more pronounced gastrointestinal and neurological manifestations, more frequently present with shock, and are at a greater risk of cardiac complications, such as ventricular dysfunction and arrhythmias. Echocardiography, ECG, cardiac biomarkers and the overall clinical phenotype are therefore important in distinguishing these overlapping conditions, although individual patients may display features of both phenotypes [59,64,66].

In children with prominent encephalopathy, seizures, movement abnormalities, psychiatric or behavioral change, cognitive deterioration, or autonomic dysfunction, autoimmune encephalitis should also be considered. Brain MRI, EEG and CSF studies should be performed according to the clinical phenotype, with neuronal autoantibody testing in serum and CSF when autoimmune encephalitis is suspected. Importantly, antibody testing should complement rather than replace clinical assessment because antibody-negative autoimmune encephalitis may occur. Similarly, acute disseminated encephalomyelitis (ADEM) should be considered in children with encephalopathy accompanied by multifocal neurological deficits, particularly when MRI demonstrates multifocal bilateral T2/FLAIR hyperintense lesions involving the cerebral white matter, deep gray structures, brainstem, cerebellum, or spinal cord [13].

Cerebrovascular and endothelial complications require urgent consideration when focal neurological deficits, abrupt impairment of consciousness, severe headache, seizures, visual disturbances, or marked hypertension are present. Ischemic or hemorrhagic stroke may occur in the setting of hypercoagulability, endothelial injury, inflammatory vasculopathy, or cardiac dysfunction, whereas headache, seizures, visual symptoms, encephalopathy and hypertension should raise suspicion of posterior reversible encephalopathy syndrome (PRES) [13,47]. Urgent MRI with diffusion-weighted sequences and vascular imaging, or CT/CT angiography when MRI is not immediately available, should be selected according to clinical urgency.

Finally, metabolic, toxic, medication-related and intensive-care-related causes of encephalopathy or delirium should be systematically considered [67,68]. Assessment should include glucose, electrolytes, renal and hepatic function, acid-base status and other metabolic investigations according to the clinical context, together with a review of potential toxic exposures. Medication reconciliation is particularly important in critically ill children because sedatives, opioids, anticholinergic agents, corticosteroids and other medications may precipitate or exacerbate altered mental status, agitation, hallucinations, sleep–wake disturbance or delirium [67]. Hypoxia, hypotension, organ failure, sleep disruption, mechanical ventilation and the intensive-care environment may additionally contribute to delirium [69]. Accordingly, not every neuropsychiatric manifestation arising during PIMS/MIS-C should automatically be interpreted as direct neuroinflammatory involvement.

**Figure 2:**
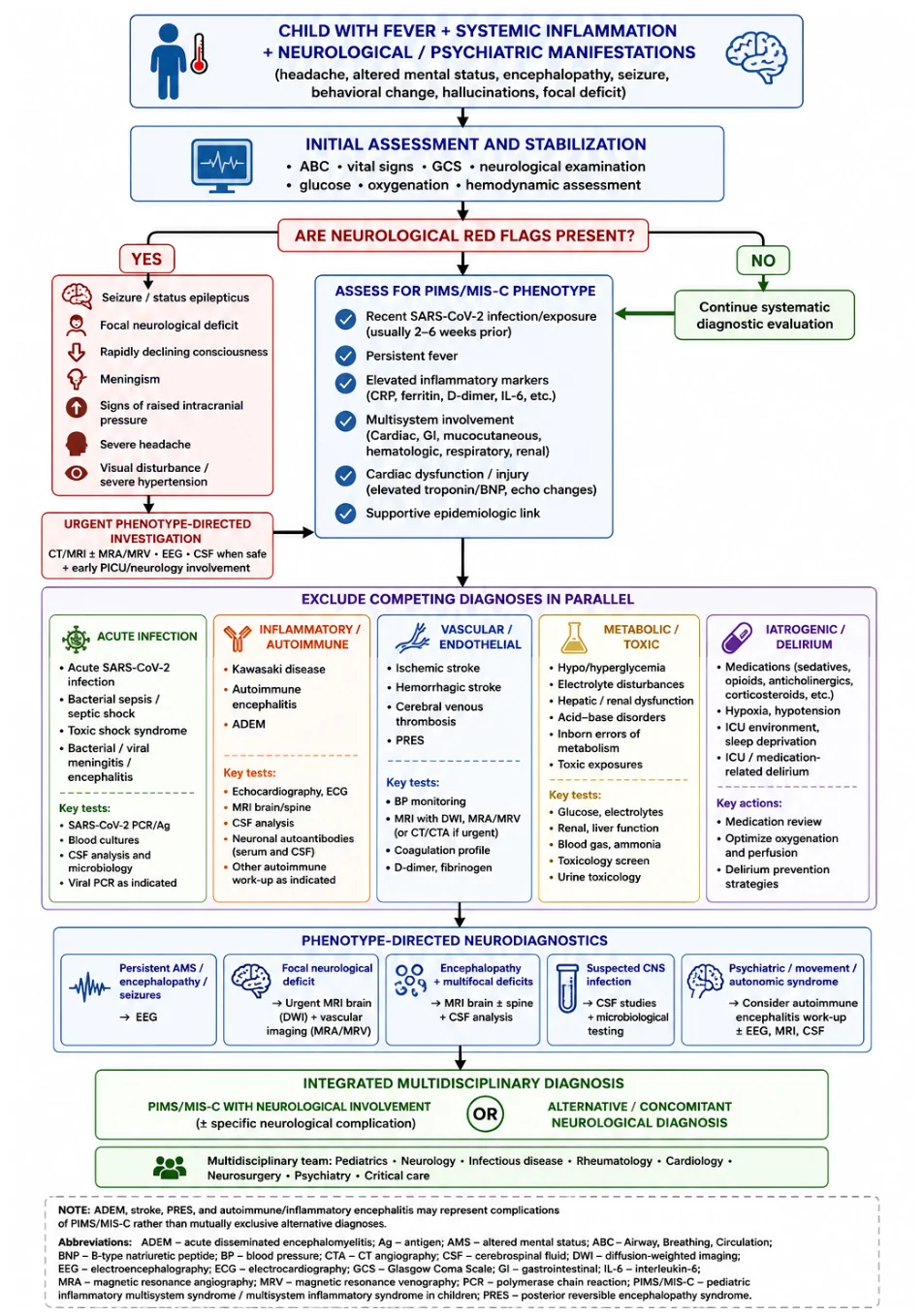
Practical diagnostic algorithm for neurological and psychiatric manifestations in suspected PIMS/MIS-C. The diagnostic evaluation should proceed in parallel, with initial stabilization and recognition of neurological emergencies followed by assessment of the systemic PIMS/MIS-C phenotype and active exclusion of competing or concomitant diagnoses. Acute SARS-CoV-2 infection, bacterial or viral CNS infection, sepsis/toxic shock syndrome, Kawasaki disease, autoimmune encephalitis, ADEM, cerebrovascular disease/PRES, metabolic or toxic encephalopathy, and medication- or intensive-care-related delirium should be considered according to the clinical phenotype. MRI and vascular imaging, EEG, CSF examination, microbiological studies, metabolic/toxic screening, and autoimmune investigations should be selected according to presenting neurological features. Importantly, ADEM, stroke, PRES, and inflammatory encephalitis may represent complications of PIMS/MIS-C rather than mutually exclusive alternative diagnoses. Abbreviations: ADEM, acute disseminated encephalomyelitis; AE, autoimmune encephalitis; AMS, altered mental status; CSF, cerebrospinal fluid; CTA, computed tomography angiography; DWI, diffusion-weighted imaging; EEG, electroencephalography; GCS, Glasgow Coma Scale; MRA, magnetic resonance angiography; MRV, magnetic resonance venography; PIMS/MIS-C, pediatric inflammatory multisystem syndrome/multisystem inflammatory syndrome in children; PRES, posterior reversible encephalopathy syndrome.

A practical diagnostic approach should therefore proceed in parallel rather than sequentially: stabilization and identification of neurological emergencies should occur simultaneously with confirmation of the systemic PIMS/MIS-C phenotype and targeted exclusion of competing infectious, inflammatory, vascular, metabolic and iatrogenic disorders (Figure 2). The subsequent neurological work-up should be guided by the predominant phenotype, with MRI and vascular imaging for focal deficits or suspected inflammatory/demyelinating disease, EEG for seizures or unexplained persistent encephalopathy, and CSF examination for suspected CNS infection or autoimmune/inflammatory encephalitis.

## 6. Neuroimaging Findings

**Figure 3:**
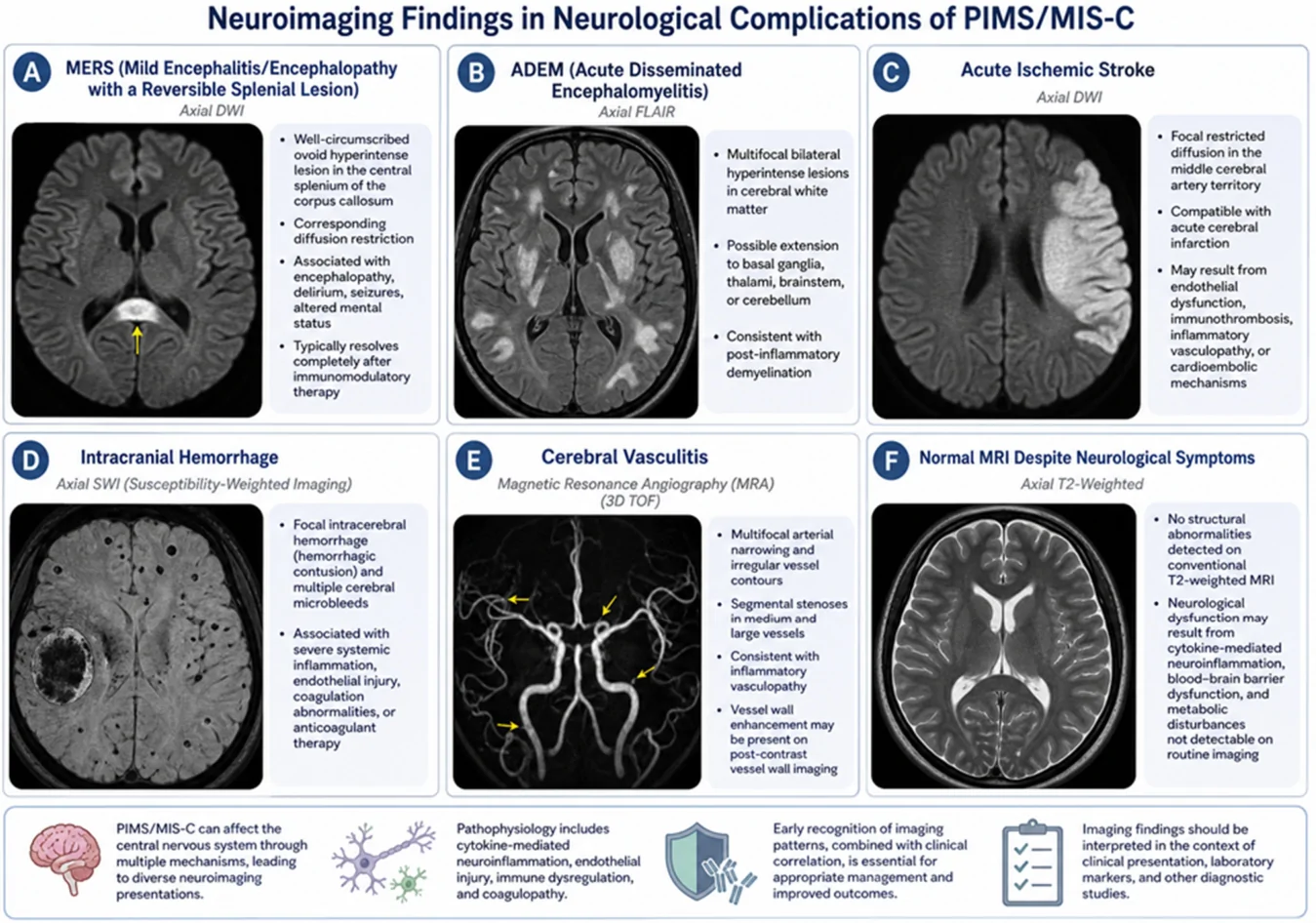
Representative magnetic resonance imaging findings in children with neurological involvement in PIMS/MIS-C.

Neuroimaging plays a central role in the evaluation of children with PIMS/MIS-C, who present with neurological symptoms. Although many patients demonstrate normal structural imaging despite significant neurological dysfunction, magnetic resonance imaging (MRI) remains the imaging modality of choice because of its superior sensitivity for detecting inflammatory, ischemic, demyelinating, and vascular abnormalities. Neuroimaging not only facilitates differential diagnosis but also provides important prognostic information and may contribute to individualized therapeutic decision-making. The radiological findings observed in PIMS/MIS-C are highly heterogeneous, reflecting the multifactorial mechanisms underlying neurological injury, including neuroinflammation, endothelial dysfunction, blood–brain barrier disruption, immunothrombosis, and immune-mediated demyelination (Figure 3).

While neuroimaging in children with PIMS/MIS-C often reveals no structural abnormalities, among the specific subset of patients who undergo magnetic resonance imaging (MRI) for severe encephalopathy and demonstrate structural abnormalities, reversible lesions involving the splenium of the corpus callosum are frequently described in case series. This radiological entity, commonly referred to as mild encephalitis/encephalopathy with a reversible splenial lesion (MERS), is characterized by a transient, well-circumscribed lesion demonstrating restricted diffusion within the central splenium on diffusion-weighted imaging (DWI), accompanied by corresponding low apparent diffusion coefficient (ADC) values [12,16,19,22,26,45,49,56].

Acute disseminated encephalomyelitis (ADEM) represents one of the most severe inflammatory neuroimaging phenotypes associated with PIMS/MIS-C. MRI typically demonstrates multiple bilateral hyperintense lesions on T2-weighted and fluid-attenuated inversion recovery (FLAIR) sequences involving the cerebral white matter, basal ganglia, thalami, brainstem, cerebellum, and occasionally the spinal cord [19]. Variable contrast enhancement and diffusion restriction may also be observed depending on lesion activity. The development of ADEM supports an autoimmune mechanism triggered by the post-infectious inflammatory response to SARS-CoV-2. Early diagnosis is essential because prompt treatment with high-dose corticosteroids, intravenous immunoglobulin, or plasma exchange is associated with improved neurological recovery and reduced long-term disability.

Although uncommon, ischemic stroke represents one of the most devastating neurological complications of PIMS/MIS-C. MRI with diffusion-weighted imaging demonstrates acute cerebral infarction corresponding to areas of restricted diffusion, while magnetic resonance angiography (MRA) may identify large-vessel occlusion or arterial narrowing. Reported infarcts involve both anterior and posterior circulation territories and may occur in association with severe cardiac dysfunction or systemic hypercoagulability [14,17,19,26,42].

Intracranial hemorrhage is considerably less common than ischemic stroke but has been reported in critically ill children with severe PIMS/MIS-C. Hemorrhagic complications may result from disseminated intravascular coagulation, thrombocytopenia, endothelial injury, anticoagulation therapy, or rupture of inflamed cerebral vessels. MRI susceptibility-weighted imaging (SWI) and computed tomography (CT) are useful for detecting intracerebral hemorrhage and cerebral microbleeds. Although rare, hemorrhagic lesions are generally associated with severe systemic disease and poorer neurological outcomes [19].

Diffuse cytokine-mediated neuronal dysfunction, microglial activation, astrocyte activation, blood–brain barrier disruption, and metabolic disturbances may impair neuronal function without producing detectable structural abnormalities on routine MRI sequences. Consequently, a normal MRI should not exclude neurological involvement or delay appropriate immunomodulatory therapy. In such cases, electroencephalography (EEG), cerebrospinal fluid analysis, advanced MRI techniques, or functional imaging may provide complementary diagnostic information.

## 7. Treatment Strategies

Treatment of PIMS/MIS-C is directed at suppressing systemic hyperinflammation, preventing cardiovascular and thrombotic complications, and providing organ-specific supportive care. Because neurological involvement often accompanies a more severe systemic phenotype, children presenting with encephalopathy, seizures, focal neurological deficits, impaired consciousness, cerebral edema, or neuroinflammatory syndromes require early multidisciplinary assessment and closer monitoring. However, the presence of mild neurological symptoms alone does not necessarily mandate a different immunomodulatory regimen; treatment intensity should reflect the overall severity of inflammation, hemodynamic instability, cardiac involvement, and the nature of the neurological complication. The manuscript’s reviewed evidence similarly indicates that neurological involvement is associated with more frequent intensive care admission and the need for coordinated neurological, cardiac, and critical-care management [48,63].

### 7.1. First-Line Systemic Management

For most hospitalized children with PIMS/MIS-C, first-line immunomodulatory treatment consists of intravenous immunoglobulin combined with systemic corticosteroids [66,70,71,72]. This dual approach is intended to achieve rapid control of the inflammatory response and reduce the risk of persistent organ dysfunction. Clinical response should be assessed through resolution of fever, improvement in hemodynamic and neurological status, and serial measurements of inflammatory and cardiac markers. Children with mild disease and no organ-threatening manifestations may occasionally be treated with IVIG alone, particularly when corticosteroids are contraindicated. Nevertheless, close observation is required because PIMS/MIS-C can deteriorate rapidly, and treatment should be intensified promptly if fever, inflammation, or organ dysfunction persists. Current American College of Rheumatology guidance recommends IVIG plus low-to-moderate-dose glucocorticoids as first-line therapy for most hospitalized patients. In accordance with these recommendations, therapeutic intensity should be stratified based on the severity of systemic inflammation, cardiac involvement, and the presence of life-threatening neurological manifestations [66].

IVIG is generally administered at a total dose of 2 g/kg, calculated using ideal body weight, with a recommended maximum dose of 100 g [66,73]. Before treatment, cardiac function and fluid status should be evaluated because patients with myocardial dysfunction or shock may be vulnerable to fluid overload. In such cases, IVIG may be infused more slowly, administered as two divided doses of 1 g/kg on consecutive days, and accompanied by diuretic therapy where appropriate.

IVIG is particularly relevant when the presentation includes Kawasaki disease-like features, coronary artery abnormalities, Guillain–Barré syndrome, or other immune-mediated peripheral neurological complications. The attached review identifies IVIG as the preferred treatment for PIMS/MIS-C-associated Guillain–Barré syndrome and as part of the therapeutic approach for severe encephalopathy, cerebral edema, and demyelinating disease. Routine administration of a second full IVIG dose in refractory PIMS/MIS-C is generally discouraged because of the risks of hemolytic anemia, excessive volume administration, and worsening cardiac overload. Failure to respond should instead prompt consideration of corticosteroid escalation or biological therapy. Low-to-moderate-dose glucocorticoids, commonly methylprednisolone or an equivalent agent at approximately 1–2 mg/kg/day, are usually administered together with IVIG [66]. Treatment should subsequently be tapered according to clinical response and normalization of inflammatory and cardiac markers; a taper lasting two to three weeks or longer may be necessary to reduce the risk of rebound inflammation. High-dose intravenous methylprednisolone pulses, usually 10–30 mg/kg/day for one to three days, may be considered in patients with refractory inflammation or life-threatening disease, including persistent shock, severe myocardial dysfunction, cerebral edema, profound encephalopathy, or inflammatory demyelination [66]. In ADEM, high-dose corticosteroids are the principal neurological treatment, with IVIG or plasma exchange considered when the response is inadequate. The dose and duration should be individualized. Children receiving high-dose corticosteroids require monitoring for hypertension, hyperglycemia, electrolyte disturbances, gastrointestinal complications, infection, sleep disturbance, agitation, delirium, and steroid-related psychiatric symptoms.

### 7.2. Treatment of Refractory PIMS/MIS-C

Refractory PIMS/MIS-C may be defined by persistent fever, continuing systemic inflammation, or ongoing significant organ dysfunction despite initial IVIG and glucocorticoid treatment. Because clinical improvement is often expected within approximately 24 h, failure to stabilize should lead to early multidisciplinary reassessment rather than repeated delays or routine re-administration of IVIG. Recommended intensification options include high-dose intravenous corticosteroids, anakinra, or infliximab.

Anakinra, a recombinant interleukin-1 receptor antagonist, is the most frequently recommended biological agent for refractory PIMS/MIS-C. It is especially appropriate in children with features of macrophage activation syndrome, persistent hyperinflammation, contraindications to prolonged corticosteroid exposure, or severe organ involvement despite initial therapy. Doses above 4 mg/kg/day, administered subcutaneously or intravenously and divided according to disease severity, have been proposed in expert guidance. Nevertheless, evidence for a specific neurological benefit is based largely on small observational series and individual reports rather than controlled trials [66,70,74,75].

Infliximab, an anti-tumor necrosis factor monoclonal antibody, may be considered as an alternative escalation agent when PIMS/MIS-C remains refractory to IVIG and glucocorticoids or when reduction in cumulative corticosteroid exposure is desirable. A single intravenous dose of approximately 5–10 mg/kg has been used in published protocols [66,76]. Infliximab should generally be avoided when macrophage activation syndrome is suspected. Screening for active or latent infection should be considered whenever clinically feasible, although urgent treatment may need to proceed before all results are available. The evidence base remains observational, and no specific advantage has been established for children with neurological involvement.

Tocilizumab, an interleukin-6 receptor inhibitor, has been used in selected cases of refractory PIMS/MIS-C, particularly when marked IL-6-driven hyperinflammation is suspected. The biological rationale is supported by the prominent role of IL-6 in systemic inflammation, endothelial activation, blood–brain barrier disruption, and neurological injury. The manuscript already identifies IL-1 and IL-6 pathways as potential therapeutic targets in refractory disease. However, tocilizumab is not generally preferred over anakinra or infliximab because experience in PIMS/MIS-C is limited, its prolonged biological effect may complicate management of concurrent infection, and suppression of C-reactive protein may obscure assessment of inflammatory activity. Its use should therefore be reserved for carefully selected refractory cases following consultation with pediatric rheumatology, infectious-disease, and intensive-care specialists [27]. Immunomodulation should be accompanied by active treatment of neurological, cardiovascular, respiratory, renal, and hematological complications. Supportive measures are not secondary to immunotherapy; in severe neuro-PIMS/MIS-C, they are often decisive for preventing irreversible injury [66]. While there is strong mechanistic plausibility suggesting that IL-1 and IL-6 blockade could mitigate blood–brain barrier disruption and targeted neuroinflammation, it must be explicitly stated that there is currently no convincing clinical evidence demonstrating that any biological agent provides a specific neurological advantage in MIS-C.

Low-dose aspirin, generally 3–5 mg/kg/day up to 81 mg/day, is recommended for many children with PIMS/MIS-C unless active bleeding, marked thrombocytopenia, or another major bleeding risk is present [66]. It is commonly continued until platelet counts normalize and normal coronary arteries are confirmed at follow-up. Therapeutic anticoagulation is indicated in children with documented thrombosis, severe ventricular dysfunction, or large coronary artery aneurysms. In patients without these indications, anticoagulation intensity should be individualized according to age, immobilization, central venous access, intensive care admission, malignancy, markedly elevated D-dimer concentrations, and bleeding risk.

### 7.3. Treatment of Specific Neurological Complications

It is important to emphasize that high-quality, prospective data regarding the optimal treatment of specific neurological and psychiatric complications in PIMS/MIS-C remain highly limited. Currently, it is not well established whether the emergence of severe neuropsychiatric symptoms warrants a distinct, neuro-specific therapeutic algorithm, or if standard systemic immunomodulation is sufficient to resolve these localized manifestations. In the absence of controlled trials comparing neuro-directed therapies in this specific pediatric population, current clinical management strategies are largely extrapolated from general MIS-C protocols, as well as established practices for other acute neuroinflammatory conditions. In a series of five COVID-19 patients presenting with psychiatric symptoms (*n* = 3), encephalopathy, and seizures (*n* = 2), immunomodulatory therapy yielded significant clinical improvement. Treatment regimens included intravenous corticosteroids alone for psychiatric symptoms (*n* = 1) and comprehensive MIS-C-like immunotherapy (including IVIG, high-dose corticosteroids, anakinra, and tocilizumab) for the remaining patients (*n* = 3). Following this targeted immunomodulation, all five patients achieved full or near-complete resolution of their neuropsychiatric symptoms by hospital discharge [77].

While standard immunomodulation addresses the underlying hyperinflammation, specific acute neurological events require targeted symptomatic management. Benzodiazepines are generally used for initial control of prolonged seizures, followed by an appropriate antiseizure medication when recurrent seizures or status epilepticus occur. The choice of agent should account for cardiac function, liver and kidney impairment, drug interactions, and hemodynamic stability [67]. Continuous or repeated EEG monitoring should be considered in children with persistent encephalopathy, fluctuating consciousness, unexplained deterioration, or suspected non-convulsive status epilepticus. Long-term antiseizure therapy is not automatically required after a single inflammation-associated seizure and should be based on seizure recurrence, EEG abnormalities, structural brain injury, and neurological follow-up.

### 7.4. Pediatric Intensive Care (PICU) Management

Admission to a pediatric intensive care unit is appropriate for children with shock, significant ventricular dysfunction, escalating vasoactive requirements, respiratory failure, severe encephalopathy, recurrent seizures, status epilepticus, cerebral edema, impaired airway protection, or rapidly progressive neurological deficits [42,44]. Management may include cautious fluid resuscitation, vasoactive or inotropic support, mechanical ventilation, correction of electrolyte and metabolic abnormalities, renal replacement therapy, invasive hemodynamic monitoring, and management of raised intracranial pressure. In suspected cerebral edema, neurocritical-care principles should include head elevation, avoidance of hypoxia and hypotension, maintenance of normoglycemia and normothermia, appropriate ventilation, urgent neuroimaging, and consideration of hyperosmolar treatment according to established pediatric neurocritical-care practice. Sedation should be regularly reviewed because medication effects may worsen or mask encephalopathy and delirium. Standardized delirium screening, sleep promotion, early mobilization, and family presence should be incorporated whenever possible. Children with neurological involvement are more likely to require mechanical ventilation and intensive care, making early PICU involvement particularly important.

### 7.5. Post-Acute Care and Neurorehabilitation

Rehabilitation should begin during hospitalization once the child is clinically stable. Early physiotherapy and occupational therapy can address weakness, deconditioning, impaired balance, neuropathy, myositis, or motor deficits following stroke, ADEM, or prolonged intensive care admission. Speech and swallowing assessment is required when bulbar dysfunction, cranial neuropathy, prolonged intubation, or cognitive-communication difficulties are present. After discharge, follow-up should be individualized according to the acute neurological phenotype. Children with persistent attention difficulties, slowed processing, memory problems, emotional dysregulation, anxiety, sleep disturbance, or school difficulties may require formal neuropsychological evaluation, psychological support, and educational accommodations. Those recovering from severe illness may develop post-intensive care syndrome involving physical, cognitive, and psychiatric domains. The paper therefore appropriately emphasizes multidisciplinary follow-up and rehabilitation for persistent neurocognitive and functional sequelae.

## 8. Neurological Prognosis and Recovery in PIMS/MIS-C

Although the acute presentation of PIMS/MIS-C can be severe, the overall prognosis is favorable, and most children recover without major permanent neurological disability. Systemic and neurological symptoms typically resolve within weeks to months after treatment. However, apparent recovery of cardiac and other organ functions does not necessarily indicate complete neurological or psychosocial restoration. A subgroup of patients experiences persistent fatigue, cognitive impairment, behavioral changes, or psychiatric symptoms that may interfere with daily functioning [48,78].

Children with severe encephalopathy, shock, prolonged ICU admission, cerebrovascular injury, or inflammatory white-matter lesions are at greater risk of protracted or incomplete recovery. While mild neurological manifestations and many reversible neuroimaging findings improve with control of inflammation, severe complications such as stroke, ADEM, encephalitis, Guillain–Barré syndrome, or prolonged status epilepticus can leave residual deficits necessitating long-term neurological observation and individualized rehabilitation. Given the high severity of the syndrome, effective primary prevention strategies remain essential to protect pediatric populations. Recent evidence underscores the critical role of vaccination in adolescents aged 12–18 years, with studies demonstrating that a 2-dose mRNA regimen provides approximately 91% effectiveness against MIS-C and prevents severe, life-threatening complications requiring intensive care [79].

Recent longitudinal evidence reinforces an optimistic outlook for the majority of children, even those requiring intensive care. In a prospective follow-up study of PICU admissions for PIMS/MIS-C, multidimensional neuropsychological testing at 24 months post-discharge demonstrated cognitive outcomes comparable to the general population norm. While self-reported functional complaints—such as fatigue (23%) or exercise intolerance (17%)—were present in a subset of patients, and a minority (14%) experienced delays in school participation, the overarching finding was one of robust recovery [80]. In the group of the seventy-five identified patients with PIMS-TS and new-onset neurological involvement, half of the patients achieved full recovery without neurological impairment at the 3-month follow-up [11]. These data suggest that while structured follow-up is essential to identify domains for targeted rehabilitation, persistent neurological or cognitive disability should not be considered an inevitable consequence of severe MIS-C.

## 9. Personalized Medicine: Current Perspectives and Future Directions

The marked heterogeneity of PIMS/MIS-C highlights the limitations of uniform diagnostic and therapeutic approaches, prompting interest in personalized medicine frameworks. Current evidence indicates that risk stratification relies primarily on conventional clinical, inflammatory, and cardiac markers, while neuroaxonal injury biomarkers (e.g., NfL, GFAP) remain strictly investigative. Integrating these emerging biomarkers with routine clinical data could theoretically help identify patients at higher risk for neurological complications and guide early immunomodulatory escalation. However, a significant evidence gap persists, particularly regarding advanced technologies such as multi-omics, genomics, and machine-learning prediction models; these tools are currently in early exploratory phases and are not ready for clinical implementation. Preliminary evidence points to a potential genetic basis for MIS-C; specifically, the HLA class I alleles A*02, B*35, and C*04 have been associated with TRBV11-2 T-cell clonal expansion and increased disease severity, although data in this area remain limited [33]. Before such approaches can influence bedside care, required validation demands rigorous, prospective testing in large multi-center cohorts to prove that they offer actionable clinical utility beyond standard evaluation. Consequently, while multi-omics and AI-driven models represent compelling future research directions, current clinical practice should instead focus on established, risk-stratified multidisciplinary follow-up—incorporating neuropsychological testing, psychiatric screening, and structured return-to-school plans for high-risk survivors.

Long-term care should be risk-stratified and multidisciplinary, incorporating neuropsychological testing, psychiatric screening, staged return-to-school plans, and multidimensional quality-of-life assessments. Table 1 outlines the primary studies reporting neurological and neuropsychiatric findings.

## 10. Study Limitations

This review has several limitations that should be acknowledged. First, as a narrative review, it does not adhere to the strict inclusion/exclusion criteria or the comprehensive quality assessment protocols required for a systematic review or meta-analysis. Consequently, the findings are subject to selection bias, as the literature included may reflect the perspectives of published case series and observational studies rather than the totality of available evidence. Second, the quality of primary studies is highly variable; many reports are limited by small sample sizes, lack of standardized diagnostic criteria for neurological and neuropsychiatric manifestations, and a frequent absence of pre-morbid baseline data. This makes it difficult to definitively distinguish between direct neuroinflammatory sequelae and secondary complications arising from critical illness, such as the trauma of intensive care or drug-induced effects. Third, the long-term neuropsychiatric outcomes remain poorly characterized, as the majority of current literature focuses on the acute or subacute phases of the syndrome. Finally, the rapid evolution of PIMS/MIS-C management protocols and the heterogeneity of patient cohorts across different geographic regions limit the generalizability of findings regarding both diagnostic biomarkers and treatment efficacy. These limitations underscore the necessity for large-scale, prospective, multicenter studies to establish rigorous evidence-based management algorithms.

## 11. Conclusions

PIMS/MIS-C is a rare but potentially severe post-infectious hyperinflammatory syndrome that can involve both the central and peripheral nervous systems. Although most children achieve good neurological recovery, a subset develops lasting cognitive, behavioral, or psychiatric difficulties. Early recognition of neurological involvement, prompt immunomodulatory therapy, and coordinated multidisciplinary management are essential to minimize morbidity.

Future progress relies on prospective multicenter studies with standardized neurological and neuropsychological evaluations, integrated biomarker research, and advanced neuroimaging. Transitioning toward precision medicine—incorporating detailed phenotyping, genetics, and computational approaches—represents a key direction for improving individualized care and long-term outcomes in children with this complex syndrome.

## Figures and Tables

**Table 1:** Summary of primary studies reporting neurological and neuropsychiatric manifestations in PIMS/MIS-C.

Author (Year)	Population, n	Study Design	Main Neurological/Neuropsychiatric Findings
Sa et al. (2021) [11]	75 PIMS-TS; 9 (12%) with neurological involvement	Single-center retrospective cohort; 3-month follow-up	Altered consciousness, behavioral changes, focal deficits, headache, hallucinations, somnolence and seizures. Neurological involvement was associated with higher CRP, procalcitonin and D-dimer. At 3 months, 4/9 had fully recovered.
Bova et al. (2022) [13]	62 MIS-C; 58 (93.5%) with neurological symptoms; 16 (26%) severe	Single-center retrospective clinical, EEG, MRI and CSF study	Altered mental status (46.7%), focal neurological signs (35.4%) and nonspecific neurological symptoms (87%). EEG slowing occurred in 20/26; corpus-callosum lesions in 3/10 MRIs. Neurological examination was normal at discharge.
Francoeur et al. (2024) [14]	3568 hospitalized children; 588 MIS-C	Prospective international multicenter cohort	Severe neurological manifestations occurred in 24.8% of MIS-C patients. New morbidity at discharge occurred in 28.0% with versus 15.5% without severe neurological manifestations (adjusted OR 2.18, 95% CI 1.22–3.89).
Wati et al. (2025) [20]	37 critically ill MIS-C patients; 12 (32.4%) with neurological manifestations	Single-center retrospective cross-sectional study	Decreased consciousness (58.3%), seizures (25%), hemiparesis (8.3%) and behavioral changes (8.3%). Neurological involvement occurred in a clinically severe PICU population.
Abbati et al. (2022) [23]	345 children: 304 acute SARS-CoV-2 and 41 MIS-C; 27/41 MIS-C with neurological/neuropsychiatric manifestations	Single-center retrospective cohort	In MIS-C: impaired consciousness (70.4%), headache (33.3%), behavioral disorders (18.5%) and mood changes (18.5%). Persistent neurological impairment was uncommon during follow-up.
Schöbi et al. (2024) [25]	75 PIMS-TS; 58 parent and 26 self-report questionnaires	Prospective 6-month mental-health follow-up	At-risk total SDQ scores occurred in 20.7% of parent reports and 26.9% of self-reports; emotional and hyperactivity/inattention difficulties were most prominent.
Sakuma et al. (2023) [31]	31 pediatric acute encephalopathy cases; 5 associated with MIS-C/PIMS	Nationwide retrospective surveillance study	Severe encephalopathy phenotypes included acute fulminant cerebral edema and other clinico-radiological syndromes; 9/31 had severe sequelae or died.
Porritt et al. (2021) [33]	20 MIS-C and 15 febrile controls	Mechanistic case–control study	TRBV11-2 T-cell expansion was concentrated in severe MIS-C and correlated with disease severity and cytokine levels; exploratory association with HLA-A02, B35 and C04.
Charlesworth et al. (2025) [36]	63 PIMS-TS	Single-center retrospective cohort	Vitamin D levels inversely correlated with peak CRP and duration of vasoactive support; associations do not establish causality or treatment benefit.
Herini et al. (2023) [41]	74 neurologically symptomatic children with SARS-CoV-2; 18 MIS-C	Single-center observational cohort	Altered mental status was more frequent in MIS-C than non-MIS-C patients (50.0% vs. 14.2%); MIS-C was also associated with longer hospitalization.
Fink et al. (2022) [42]	1493 hospitalized children; 215 MIS-C	International multicenter cross-sectional study	Neurological manifestations occurred in 66% of MIS-C versus 40% of acute SARS-CoV-2 cases. Headache occurred in 47% and acute encephalopathy in 22% of MIS-C. Neurological involvement was associated with ICU admission.
Ray et al. (2021) [44]	52 neurological cases; 25 PIMS-TS	Prospective UK national surveillance cohort	In PIMS-TS: encephalopathy 88%, peripheral nervous-system involvement 40%, behavioral changes 36%, hallucinations 24%; 80% required PICU admission.
Kalın & Sözeri (2022) [45]	110 MIS-C; 6 with neuroimaging abnormalities	Single-center retrospective study	Reported abnormalities included reversible splenial lesion syndrome, PRES, meningitis and cerebral edema; no pathognomonic imaging pattern was identified.
Lindan et al. (2021) [56]	38 children with SARS-CoV-2-related neurological disease and abnormal neuroimaging	Multinational referral-based case series	ADEM-like changes, myelitis, neural enhancement and splenial lesions were reported; splenial lesions occurred predominantly in MIS-C.
LaRovere et al. (2021) [65]	1695 hospitalized children; 616 MIS-C; 365 with neurological involvement	Multicenter registry-based case series	Most neurological manifestations were transient, but 43 children had life-threatening neurological disease, including severe encephalopathy, stroke, CNS infection/demyelination, GBS and cerebral edema.

Abbreviations. ADEM, acute disseminated encephalomyelitis; CI, confidence interval; CNS, central nervous system; CRP, C-reactive protein; CSF, cerebrospinal fluid; EEG, electroencephalography; HLA, human leukocyte antigen; GBS, Guillain-Barré syndrome; MIS-C, multisystem inflammatory syndrome in children; MRI, magnetic resonance imaging; OR, odds ratio; PICU, pediatric intensive care unit; PIMS-TS, pediatric inflammatory multisystem syndrome temporally associated with SARS-CoV-2; PRES, posterior reversible encephalopathy syndrome; SDQ, Strengths and Difficulties Questionnaire.

## Data Availability

No new data were created or analyzed in this study. Data sharing is not applicable to this article.

## Abbreviations

PIMS	Pediatric Inflammatory Multisystem Syndrome
MIS-C	Multisystem Inflammatory Syndrome in Children
SARS-CoV-2	Severe Acute Respiratory Syndrome Coronavirus 2
IL	Interleukin
TNF-α	Tumor Necrosis Factor-alpha
HLA	Human Leukocyte Antigen
ADEM	Acute Disseminated Encephalomyelitis
PICU	Pediatric Intensive Care Unit
EEG	Electroencephalography
PCR	Polymerase Chain Reaction
MRI	Magnetic Resonance Imaging
PRES	Posterior Reversible Encephalopathy Syndrome
GBS	Guillain–Barré Syndrome
PICS	Post-Intensive Care Syndrome
CRP	C-Reactive Protein
BNP	B-Type Natriuretic Peptide
NT-proBNP	N-Terminal Pro-B-Type Natriuretic Peptide
NfL	Neurofilament Light Chain
GFAP	Glial Fibrillary Acidic Protein
NSE	Neuron-Specific Enolase
S100B	S100 Calcium-Binding Protein B
MERS	Mild Encephalitis/Encephalopathy with a Reversible Splenial Lesion
DWI	Diffusion-Weighted Imaging
ADC	Apparent Diffusion Coefficient
FLAIR	Fluid-Attenuated Inversion Recovery
MRA	Magnetic Resonance Angiography
SWI	Susceptibility-Weighted Imaging
CT	Computed Tomography
IVIG	Intravenous Immunoglobulin
PET	Positron Emission Tomography
